# Anti-HMGB1 Neutralizing Antibody Ameliorates Neutrophilic Airway Inflammation by Suppressing Dendritic Cell-Mediated Th17 Polarization

**DOI:** 10.1155/2014/257930

**Published:** 2014-05-15

**Authors:** Fang Zhang, Gang Huang, Bo Hu, Li-Ping Fang, E-Hong Cao, Xiao-Feng Xin, Yong Song, Yi Shi

**Affiliations:** ^1^Department of Pulmonary Medicine, Jinling Hospital, Nanjing University School of Medicine, Nanjing 210002, China; ^2^Department of Medical Genetics, Third Military Medical University, Chongqing 430038, China; ^3^Department of Administration, 105 Hospital, Hefei 230031, China

## Abstract

We demonstrate that high mobility group box 1 protein (HMGB1) directs Th17 skewing by regulating dendritic cell (DC) function. First, our* in vitro* studies reveal that recombinant HMGB1 (rHMGB1) activates myeloid DCs to produce IL-23* in vitro*, and rHMGB1-activated DCs prime naïve lymphocytes to produce the Th17 cytokine IL-17A. Second, we demonstrate that anti-HMGB1 neutralizing antibody attenuates HMGB1 expression, neutrophilic inflammation, airway hyperresponsiveness, and Th17-related cytokine secretion* in vivo* by using a murine model of neutrophilic asthma induced by ovalbumin (OVA) plus lipopolysaccharide (LPS). Furthermore, anti-HMGB1 neutralizing antibody decreases the number of Th17 cells in lung cells and suppresses the production of IL-23 by lung CD11C^+^ APCs. Finally, we show that intranasal adoptive transfer of rHMGB1-activated DCs was sufficient to restore lung neutrophilic inflammation and the Th17 response in a DC-driven model of asthma, whereas the transfer of rHMGB1 plus anti-HMGB1-treated mDCs significantly reduced these inflammation phenotypes. These data suggest, for the first time, that HMGB1 drives the DC-polarized Th17-type response in allergic lung inflammation and that blocking HMGB1 may benefit the attenuation of neutrophilic airway inflammation in asthma.

## 1. Introduction

Asthma is a chronic inflammatory disease of the airways that is hypothetically considered as a T helper type 2 cell- (Th2-) mediated process [1]. However, recent studies demonstrated that T-helper type 17 cell (Th17) immune responses also play roles in pathogenesis [2]. Th17 cells are a distinct subset of T cells that have been found to produce interleukin-17 (IL-17) and differ in function from other T cell subsets, such as Th1, Th2, and regulatory T cells (Treg) [3]. For example, Th2 cells and their cytokines primarily predominate in mild to moderate allergic asthma, whereas mixed Th2 cells with a Th17 phenotype mediate severe steroid-resistant airway inflammation [4]. Furthermore, passive transfer of Ag-specific Th17 cells induced neutrophilic inflammation and airway hyperresponsiveness [5]. Th17 cells are characterized by producing IL-17 (viz. IL-17A), which is thought to cause severe asthma and induce neutrophilic inflammation [6]. However, Th17 function in severe asthma as a driving mechanism of neutrophilic inflammation is not yet fully understood and requires further exploration.

Dendritic cells (DCs) are considered the most important APCs in the initiation of the Ag-induced immune response [7]. The interaction between DCs and T cells and the existence of polarized cytokines are crucial for determining the direction of T cell polarization, such as Th1, Th2, Treg, or Th17 [8, 9]. The polarization of Th17 cells is mainly regulated by the key polarized cytokines IL-23 and IL-6 [10]. High mobility group box 1 (HMGB1) is a DNA-binding, nuclear protein that can act as an alarmin, which is a danger signal that alerts the innate immune system to initiate the host defense [11]. Some studies demonstrate that HMGB1 stimulates DCs to induce the production of Th17 polarization-related factors* in vitro* and promotes the Th17 response in acute allograft rejection [12], experimental autoimmune myocarditis [13], and rheumatoid arthritis [14]. In our recent studies, an increase in HMGB1 expression and Th17-mediated (or Th17-involved) airway inflammation have been found in a murine model of neutrophilic asthma, and HMGB1 expression in lung tissue was positively correlated with the IL-17 level or neutrophil numbers in bronchoalveolar lavage fluid (BALF) [15]. We hypothesize that HMGB1 directs Th17 skewing by regulating DC function, and HMGB1 blocking inhibits the Th17 response and Th17-mediated neutrophilic airway inflammation in asthma.

To test this hypothesis, a mouse model of neutrophilic asthma was generated using intranasal sensitization with lipopolysaccharide (LPS) plus ovalbumin (OVA) and then challenging with OVA alone. Because it is known that neutrophilic inflammation in airways is associated with bacterial LPS in the lungs of asthmatic patients [16], this model may be useful for investigating the mechanisms of Th17-mediated neutrophilic airway inflammation. The aim of this study was to evaluate whether HMGB1 promotes the Th17 response that is mediated by DCs* in vitro* and if blocking of HMGB1 inhibits the Th17 response and neutrophilic airway inflammation* in vivo*. To demonstrate the important role of HMGB1 signaling in DC-primed allergic disease, we also investigated whether adoptive transfer of HMGB1-activated DCs was sufficient to restore neutrophilic inflammation and the Th17 response in a DC-driven model of asthma. This study will uncover the immunopathological mechanisms for Th17-mediated, neutrophilic airway inflammation that is induced by HMGB1 in neutrophilic asthma.

## 2. Materials and Methods

### 2.1. Mice

Female Balb/C mice were obtained from the animal center of Jinling Hospital and maintained in a pathogen-free-authorized facility, where the temperature was maintained at 20–22°C and the humidity was kept at 50–60%. The dark/light cycles were 12 h. All animal experiments were approved by and performed in compliance with the guidelines of the Institutional Animal Care and Use Committee.

### 2.2. Culture and Challenge of DCs

Mouse bone marrow-derived DCs (BMDCs) were generated as previously described [17]. Briefly, murine femurs and tibiae were isolated mechanically from surrounding tissues, and BM cells were flushed with medium using a syringe with a 0.45 mm needle. After centrifuging the cells and lysing the RBCs using ACK, 2 × 10^5^ cells were cultured in complete RPMI 1640 medium in the presence of 10 ng/mL GM-CSF and 1 ng/mL IL-4. After 8 days of culture, DCs were purified using anti-CD11c-coated microbeads (Miltenyi-Biotec, Auburn, CA, USA) and treated with medium in the absence or presence of various concentrations of recombinant HMGB1 (rHMGB1) (10, 100, 500, or 1000 ng/mL) (R&D Systems, Minneapolis, MN) for 2 d, or with 500 ng/mL rHMGB1 stimulation for different times (1, 2, 4, and 6 d). rHMGB1 reagent was endotoxin tested with Limulus amebocyte lysate (ZhanJiang A&C Biological, China) and contained 0.003 EU/mg endotoxin content. Thus, this product is considered to be endotoxin free. Also, rHMGB1 was in the disulfide form in this study. Cytokine concentrations in the cell culture supernatants were measured using enzyme-linked immunosorbent assay (ELISA) kits (IL-23 and IL-6; R&D Systems, Minneapolis, MN).

### 2.3. Coculture of DCs with CD4^+^ T Cells

CD4^+^ T cells were purified from the spleens of mice using a “Mouse CD4^+^ T cell enrichment kit” (Stem Cell Technologies, Vancouver, BC, Canada) following the manufacturer's instructions. The purity was greater than 98%. The CD4^+^ T cells were then collected after incubation with anti-CD3 (2 *μ*g/mL) and soluble anti-CD28 (1 *μ*g/mL) in complete RPMI 1640 for 3 days and plated at 1 × 10^5^ cells/well alone or cocultured with 2.5 × 10^4^ autologous DCs or rHMGB1-stimulated DCs. In Transwell assays, the cells were separated by a membrane (6.5 mm diameter, 0.4 *μ*m pore size) in 24-well plates (Costar, Cambridge, MA). The lower compartment of the wells contained rHMGB1-stimulated DCs. The upper compartments contained anti-CD3 and anti-CD28 activated CD4^+^ T cells. When indicated, neutralizing Abs for IL-23p19 or control IgG (both from R&D Systems) was added into the coculture medium at a concentration of 0.8 *μ*g/mL. After 5 d of incubation, the culture supernatants were analyzed for IL-17A by ELISA, and the expression of intracellular IL-17 by CD4^+^ T cells was analyzed using a FACSCalibur.

### 2.4. Protocols for Mouse Model and Pharmacologic Intervention

A murine model of neutrophilic asthma that was characterized by Th17 cell responses was generated as described previously [18]. In brief, mice were intranasally sensitized with 75 *μ*g LPS-depleted OVA (grade V; Sigma-Aldrich, St. Louis, MO, USA) plus 10 *μ*g LPS (*E. coli* serotype 026 : B6; Sigma-Aldrich) on days 0, 1, 2, and 7 and then challenged with 50 *μ*g OVA alone on days 14, 15, 21, and 22. One day after the final challenge, the mice were euthanized for further analyses.

**Figure 1:**
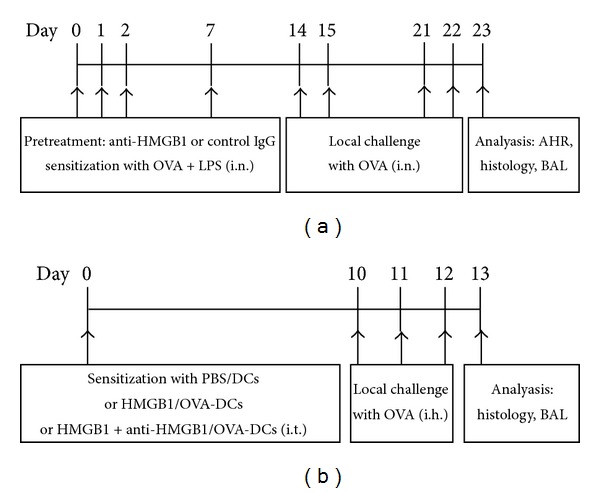
(a) OVA and LPS-induced murine model of neutrophilic asthma. Mice were intranasally sensitized with OVA plus LPS on days 0, 1, 2, and 7 and then challenged with OVA on days 14, 15, 21, and 22. Anti-HMGB1 IgG or control IgG was intranasally administered a half an hour before each OVA challenge. One day after the final challenge, the mice were euthanized for further analyses. (b) A murine model of asthma by the transfer of DCs. Mice were intratracheally sensitized with PBS/DCs, HMGB1/OVA-DCs, or HMGB1 + anti-HMGB1/OVA-DCs on day 0 and then challenged with OVA on days 10, 11, and 12. One day after the final challenge, the mice were euthanized for further analyses.

In this study, the mice were divided randomly into four groups (*n* = 6 mice) as follows: (i) mice sensitized with phosphate-buffered saline (PBS) and challenged with OVA (control group); (ii) mice sensitized with OVA plus LPS and challenged with OVA (asthma group); (iii) mice treated with control IgG (R&D Systems) for half an hour before sensitization to OVA plus LPS and the same challenge with OVA later (control IgG group); (iv) mice treated with anti-HMGB1 IgG (R&D Systems) half an hour before sensitization to OVA plus LPS and the same challenge with OVA later (anti-HMGB1 group). Anti-HMGB1 IgG or control IgG was administered intranasally (200 *μ*g/kg) on days 14, 15, 21, and 22 a half an hour before OVA challenge (Figure 1(a)). The dose of anti-HMGB1 IgG was predetermined using staining analysis of airway inflammation in mice receiving 100–400 *μ*g/kg of anti-HMGB1 IgG.

### 2.5. Bronchoalveolar Lavage Fluid (BALF)

One day after the challenges, the mice were anesthetized, and their BALF was collected as described previously [19]. The cells in the BALF samples were centrifuged and loaded onto slides. Differential cell counts were ascertained in duplicate on coded slides using 200 cells from each sample. The levels of IL-23, IL-17A, and IL-4 and interferon-gamma (IFN-*γ*) in the BALF were determined using ELISA according to the manufacturer's instructions (eBioscience).

### 2.6. Histological Examination

After the mice were euthanized, their lungs were fixed in 4% buffered formalin, and lung tissue sections were stained with hematoxylin and eosin (H&E). The sections were observed using a microscope at ×200 magnification.

### 2.7. Measurement of HMGB-1 Expression

Five-micrometer-thick serial sections from the paraffin-embedded lung tissue were stained to detect the expression of HMGB-1 in the lungs as described previously [20]. Briefly, the paraffin-embedded sections were stained with primary antibodies to HMGB-1 (Santa Cruz Biotechnology Inc.) that was diluted at 1 : 50 and biotinylated secondary antibody that was diluted at 1 : 500. The bound peroxidase was visualized using the DAB method. We counted 10 randomly chosen fields per section using a microscope at ×200 magnification, and the intensity of HMGB-1 protein staining was determined as the average optical density using IPP software (Image-Pro Plus 6.0, Media, Cybernetics). A nonstained region was selected and set as the background.

### 2.8. Airway Hyperreactivity (AHR)

AHR was analyzed 24 h after the last OVA aerosol challenge. To analyze the AHR, methacholine-induced airflow obstructions from conscious mice that were placed in a whole body plethysmograph (model PLY 3211, Buxco Electronics, Troy, NY, USA) were performed, and the dimensionless parameter known as enhanced pause (Penh) was measured as previously described [19]. Briefly, the mice were placed in the plethysmograph chamber and exposed to aerosolized, normal saline solution (baseline readings) and then to cumulative concentrations of *β*-methacholine that ranged from 3 to 24 mg/mL. The aerosol was generated using an ultrasonic nebulizer and drawn through the chamber for 2 minutes. The inlet was then closed, and Penh readings were taken for 3 minutes and averaged. The values were then reported as the Penh index.

### 2.9. Flow Cytometry Analysis

To detect IL-17^+^ CD4^+^ T cells in the DC-T cell coculture system, the cells were incubated with 10 *μ*g/mL brefeldin A (eBioscience) for 2 h and stained with FITC-anti-CD4 mAb (eBioscience) for 30 min at 4°C. After incubation with the fixation/permeabilization solution (eBioscience), the cells were stained with PE-anti-IL-17 mAb (eBioscience) for 30 min and analyzed using a FACSCalibur flow cytometer (BD Biosciences) and the CellQuest software.

To detect IL-17^+^ CD4^+^ T cells in lung tissue, lung cells were obtained according to previously reported methods [21]. Briefly, the lungs were minced into small pieces and incubated in a solution containing 2 mg/mL collagenase XI and 100 *μ*g/mL DNase I (both from Roche Applied Science, Indianapolis, USA) in RPMI 1640 at 37°C for 60 min. EDTA (2 mM, pH 7.2) was added during the last 5 min of incubation. Digested cells were then filtered through 70-*μ*m cell strainers, and erythrocytes were lysed using ACK lysing buffer (150 mM NH_4_Cl, 10 mM KHCO_3_, and 0.1 mM EDTA). Lung cells (4 × 10^6^/mL) were incubated with brefeldin A (10 *μ*g/mL) for 2 h and stained with surface-specific Abs (anti-CD3-APC and anti-CD4-FITC; eBioscience) for 30 min at 4°C. After incubation with the fixation/permeabilization solution (eBioscience), the cells were stained with PE-anti-IL-17 mAb (eBioscience) for 30 min and analyzed using a FACSCalibur flow cytometer.

To detect IL-23^+^ CD11c^+^ APCs in lung tissue, low-density lung cells were prepared as described previously [22]. Briefly, digested lung cells (10 × 10^6^/mL) were resuspended in high-density Percoll (*ρ* = 1.075 g/mL), overlaid with an equal volume of lower density Percoll (*ρ* = 1.030 g/mL), and centrifuged at 400 ×g for 20 min. Low-density lung cells, which were enriched for mononuclear cells, were recovered from the 1.075/1.030 Percoll interface and washed with Hanks' balanced salt solution (HBSS). Low-density lung cells were then stained with DC-specific Ab (FITC-anti-CD11C mAb, eBioscience) for 30 min at 4°C. After incubation with the fixation/permeabilization solution (eBioscience), the cells were stained with PE-anti-IL-23 mAb (eBioscience) for 30 min and analyzed using a FACSCalibur flow cytometer.

### 2.10. Adoptive Transfer of DCs

The establishment of a murine model of asthma through the transfer of bone marrow-derived DCs (BMDCs) has been described [17]. Briefly, BMDCs (cultured for 8 days) were purified and pulsed with OVA overnight (OVA-DCs). OVA-DCs were then injected into anesthetized, naïve mice via the intratracheal route. Ten days after intra-tracheal immunization, the mice were challenged with OVA (1% w/v in PBS; grade V; Sigma-Aldrich) aerosol during a daily, 30-min challenge on 3 consecutive days. In this experiments, the mice were divided randomly into the three groups (*n* = 6 per group) as follows: (i) mice received an i.t. injection of 2 × 10^6^ PBS-treated and nonpulsed DCs (PBS/DCs); (ii) mice received an i.t. injection of 2 × 10^6^ rHMGB1-treated OVA-DCs (rHMGB1/OVA-DCs); or (iii) mice received an i.t. injection of 2 × 10^6^ rHMGB1 plus anti-HMGB1-treated OVA-DCs (rHMGB1 + anti-HMGB1/OVA-DCs). The OVA-DCs were treated with rHMGB1, anti-HMGB1, or control IgG at concentrations of 500 ng/mL for 2 d. All mice were euthanized 24 h after the final challenge for further analyses (Figure 1(b)).

### 2.11. Statistical Analysis

Data were expressed as the means ± SD. Differences between groups were analyzed using SPSS for windows (version 16.0) and unpaired, two-tailed, parametric Student's *t*-test, or ANOVA tests. *P* values < 0.05 were considered statistically significant.

## 3. Results

### 3.1. rHMGB1-Stimulated BMDCs Induce the Th17 Response via IL-23* In Vitro*

**Figure 2:**
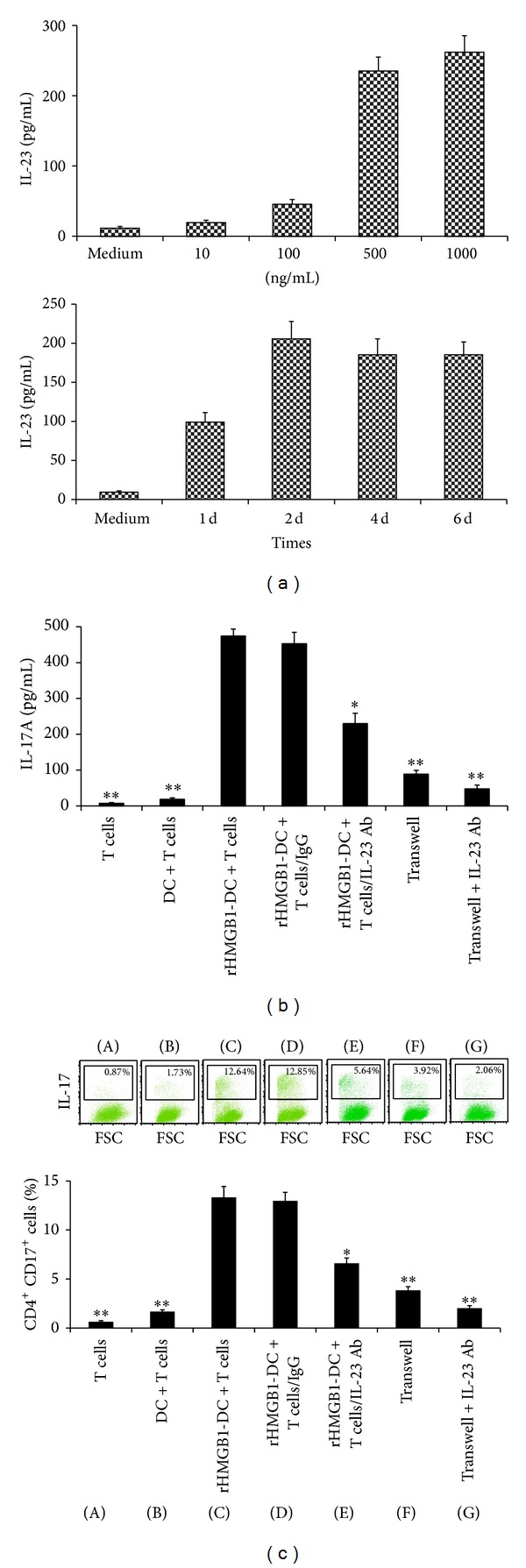
HMGB1-stimulated BMDCs induce IL-17A production and Th17 differentiation via IL-23* in vitro*. (a) HMGB1-induced IL-23 expression in BMDCs. BMDCs were stimulated with graded concentrations of HMGB1 or culture medium for 2 d or stimulated with 500 ng/mL HMGB1 for the indicated times. The IL-23 levels in the cell culture supernatants were measured using ELISA. The values represent the means ± standard deviations (*n* = 5). **P < 0.01 compared with the medium control. (b and c) HMGB1-stimulated BMDCs modulate Th17 polarization. Activated, autologous CD4^+^ T cells (1 × 10^5^) were cultured alone or cocultured with BMDCs (2.5 × 10^4^) that were treated with or without 500 ng/mL HMGB1 in the presence or absence of IL-23p19 Ab or control IgG for 5 d, the culture supernatants were analyzed for IL-17A using ELISA (b), and the expression of intracellular IL-17 by CD4^+^ T cells was analyzed using FACS (c). The values represent the means ± standard deviations (*n* = 5). **P < 0.01 compared with the HMGB1-DC group.

Because BMDCs express receptors for HMGB1, namely, RAGE (the receptor for advanced glycation end products), we tested whether HMGB1 could induce the production of Th17 polarization-related factors in these cells. We initially exposed BMDCs to various concentrations of rHMGB1 and measured their capacity to express IL-23 and IL-6 in culture supernatants. As shown in Figure 1(a), rHMGB1 increased the IL-23 levels in a 2-d culture, with significant effects at rHMGB1 concentrations of 500 ng/mL and 1000 ng/mL (Figure 2(a)). An increased tendency for IL-6 expression was observed, but it did not reach statistical significance (data not shown). Additionally, IL-23 in culture supernatants maintained a high level at treatment with a concentration of 500 ng/mL rHMGB1 for 6 d (Figure 2(a)). The results indicated that rHMGB1-stimulated DCs have the potential to secrete Th17 polarization-related factors, such as IL-23,* in vitro*.

Because antigen-presenting cells such as DCs can modulate T helper cell polarization, we investigated whether rHMGB1 stimulation of BMDCs could induce the development of a Th17 phenotype in cocultured, autologous CD4^+^ T cells. Following their 8-day culture, BMDCs were treated with 500 ng/mL rHMGB1 and cocultured with activated, autologous T cells for an additional 5-day period at a ratio of 1 : 4. The culture supernatants and T cells were then analyzed for IL-17 expression using ELISA and flow cytometry, respectively. The results showed that rHMGB1-stimulated BMDCs significantly enhanced IL-17A secretion in the culture supernatants (Figure 2(b)) and increased the percentage of IL-17^+^ CD4^+^ T cells (Figure 2(c)). In contrast, activated CD4^+^ T cells failed to upregulate their expression of IL-17 after coculturing with BMDCs. Interestingly, addition of the IL-23 antibody to the coculture significantly lowered the IL-17A expression level (Figure 2(b)) and the percentage of IL-17^+^ CD4^+^ T cells (Figure 2(c)) that were induced by the rHMGB1-stimulated BMDCs, suggesting that the IL-17 expression level was dependent upon rHMGB1 and potentially regulated by the endogenous production of IL-23 by BMDCs. In Transwell assays, the rHMGB1-stimulated BMDCs were cultured in the lower chamber, and anti-CD3 and anti-CD28 activated CD4^+^ T cells were placed in the upper chamber. The results showed that the IL-17A expression level (Figure 2(b)) and the percentage of IL-17^+^ CD4^+^ T cells (Figure 2(c)) were still mildly elevated, and significantly lowered after the addition of the IL-23 antibody to the coculture medium, suggesting that direct contact between the rHMGB1-stimulated BMDCs and preactivated T cells is not absolutely necessary, while the endogenous production of IL-23 is required.

### 3.2. Anti-HMGB1 IgG Inhibits HMGB1 Expression* In Vivo*

**Figure 3:**
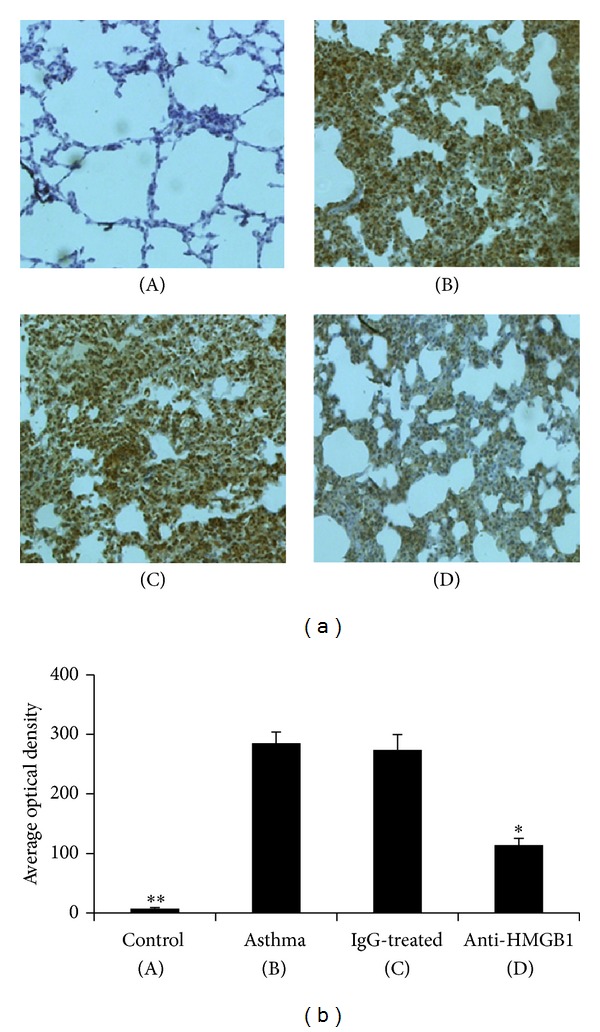
Intranasal administration of anti-HMGB1 IgG inhibited HMGB1 expression in the lung. (a) Representative images of immunohistochemical staining for HMGB1 in lung tissue from mice in the control (A), asthma (B), control IgG-treated (C), and anti-HMGB1-treated groups (D). (b) The average optical density in the histogram represents the HMGB1 expression. The values represent the means ± standard deviations (*n* = 5). *P < 0.05 and **P < 0.01 compared with the asthma group.

Because HMGB1, which is a DNA-binding factor that acts as a late inflammatory mediator, plays a major role in the maintenance of airway inflammation [23], we supposed that anti-HMGB1 IgG could decrease airway inflammation via blocking HMGB1 signaling. It was found that asthma group mice developed a significant increase in HMGB1 expression compared with control group mice (*P* < 0.01, Figure 3). To our surprise, the administration of anti-HMGB1 IgG resulted in a significant inhibition of HMGB1 expression compared with IgG-treated mice (*P* < 0.05, Figure 3), suggesting that anti-HMGB1 IgG could be an effective agent for inhibiting HMGB1 expression.

### 3.3. Anti-HMGB-1 IgG Suppresses Neutrophilic Airway Inflammation

**Figure 4:**
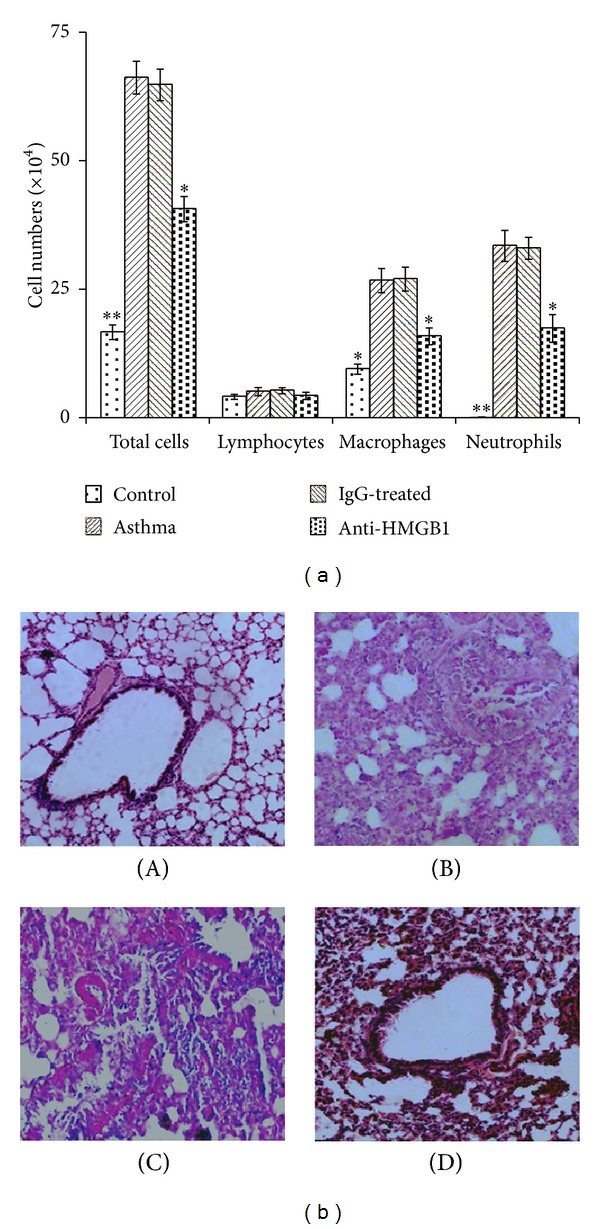
Intranasal administration of anti-HMGB1 IgG suppressed neutrophilic airway inflammation. (a) Administration of anti-HMGB1 suppressed the infiltration of neutrophils and macrophages into BALF. BALF was collected 24 hours after the last OVA challenge. The number of total and differential cells counts in the BALF of mice in the control, asthma, control IgG-treated, and anti-HMGB1-treated groups were analyzed by counting. The values represent the means ± standard deviations (*n* = 5). *P < 0.05 and **P < 0.01 compared with the asthma group. (b) Administration of anti-HMGB1 reduced the neutrophilic inflammation in lung tissue. Histological findings of lung tissues (original magnification, ×200) in mice from the control (A), asthma (B), control IgG-treated (C), and anti-HMGB1-treated groups (D) were analyzed using H&E staining.

To ascertain if anti-HMGB1 IgG could prevent neutrophilic airway inflammation, HMGB1 Abs were administered intranasally (200 *μ*g/kg) into mice before OVA sensitization. As expected, asthma group mice exhibited significantly increased counts of total cells, neutrophils, and macrophages in their BALF compared with that of the control group mice (*P* < 0.05 or 0.01, Figure 4(a)). However, administration of anti-HMGB1 IgG before sensitization resulted in a decrease in the total number of BALF cells, neutrophils and macrophages compared with IgG-treated mice (*P* < 0.05, Figure 4(a)).

We also compared changes in lung pathology in the different groups of mice. As shown in Figure 4(b), asthma group mice showed prominent neutrophil infiltration of the peribronchiolar and perivascular connective tissues, together with the hypersecretion of mucus by epithelial cells. In contrast, the administration of anti-HMGB1 IgG markedly inhibited neutrophil infiltration and mucus hypersecretion into the airways. All of these results indicated that local blockage of HMGB1 signaling by anti-HMGB1 IgG before sensitization significantly reduced neutrophil infiltration.

### 3.4. Anti-HMGB1 IgG Inhibits the Development of AHR

**Figure 5:**
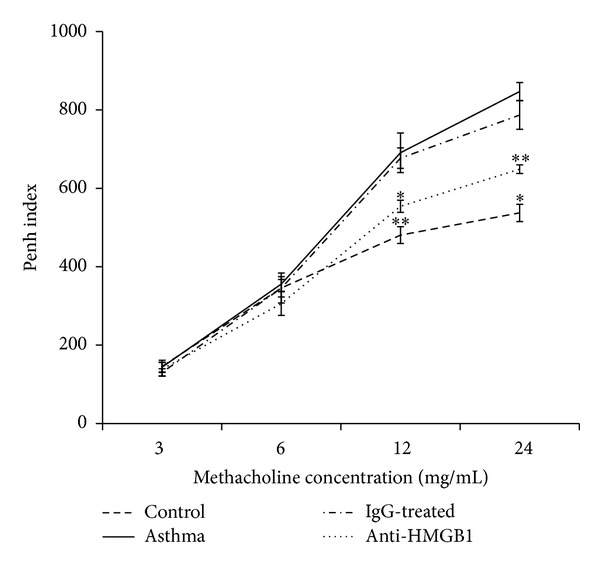
Intranasal administration of anti-HMGB1 reduced AHR to methacholine stimuli. The Penh values represent the OVA-induced AHR to methacholine and were measured in mice from the control, asthma, control IgG-treated, and anti-HMGB1-treated groups using a body plethysmograph. The values represent the means ± standard deviations (*n* = 5). *P < 0.05 and **P < 0.01 compared with the asthma group.

Next, we analyzed if administration of anti-HMGB1 IgG was important for changes in lung function. The results showed that asthma group mice developed significant AHR compared with the control group mice (*P* < 0.01) after the methacholine concentration reached 6 mg/mL (Figure 5). However, the Penh was significantly reduced in anti-HMGB1 IgG-treated mice compared with asthma group animals (*P* < 0.05, Figure 5). These results indicated that administration of anti-HMGB1 IgG could significantly inhibit the development of AHR in the murine model of neutrophilic asthma.

### 3.5. Anti-HMGB1 IgG Attenuates the Th17 Response

**Figure 6:**
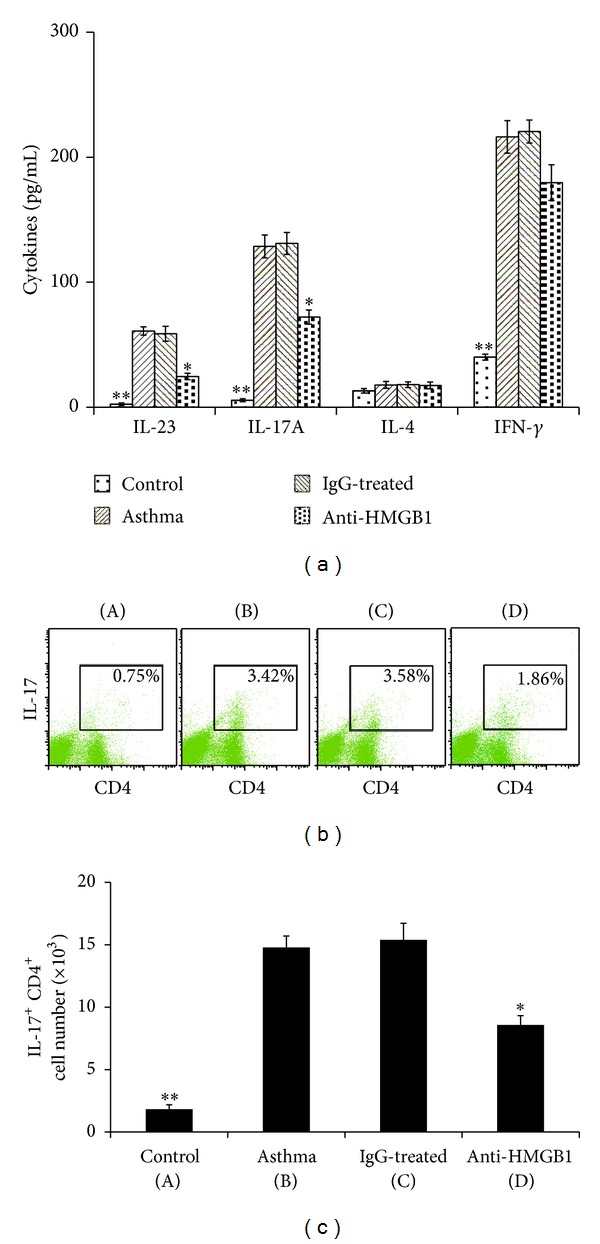
Intranasal administration of anti-HMGB1 decreased the Th17 response in the lung. (a) Administration of anti-HMGB1 decreased Th17 cytokine secretion after OVA challenge. BALFs were collected 24 hours after the last OVA challenge. The production of IL-23, IL-17A, IL-4, and IFN-*γ* in the BALF of mice in the control, asthma, control IgG-treated, and anti-HMGB1-treated groups was assessed using ELISA. The values represent the means ± standard deviations (*n* = 5). *P < 0.05 and **P < 0.01 compared with the asthma group. (b) Administration of anti-HMGB1 downregulated the number of Th17 cells in the lung. Representative results of the percentage of Th17 cells in CD3^+^ gated lung cells from mice in the control (A), asthma (B), control IgG-treated (C), and anti-HMGB1-treated groups (D) were analyzed using FACS. (c) Histogram showing the number of Th17 cells in mice from the different groups. The values represent the means ± standard deviations (*n* = 5). *P < 0.05 and **P < 0.01 compared with the asthma group.

Furthermore, we analyzed if administration of anti-HMGB1 IgG resulted in attenuation of the Th17 response. As shown in Figure 6(a), local blockage of HMGB1 led to marked reduced levels of IL-23 and IL-17A (Th17-associated cytokines) in the BALF (*P* < 0.05 versus Asthma group or IgG group). In contrast, anti-HMGB1 IgG treatment decreased the level of IFN-*γ* (Th1-associated cytokine) in the BALF, but this did not reach statistical significance (*P* = 0.064 versus Asthma group). The level of IL-4 (Th2-associated cytokine) was not affected by anti-HMGB1 IgG treatment.

Because the level of IL-17A in BALF was increased in the asthma group mice and anti-HMGB1 IgG treatment suppressed it, we examined the possibility that this suppression was mediated through the decreased production of Th17 cells in the lung. The results indicated that the number of IL-17^+^ CD4^+^ T cells (Th17) in the lung tissue was significantly increased in asthma group mice compared with control group mice (*P* < 0.01, Figure 6(b)). As expected, the administration of anti-HMGB1 IgG significantly downregulated the number of Th17 cells in lung tissue (*P* < 0.05 versus the Asthma group or IgG group, Figure 6(b)). These results suggested that anti-HMGB1 IgG could suppress the development of Th17 cells* in vivo*.

### 3.6. Anti-HMGB1 IgG Suppresses IL-23 Production by Lung CD11c^+^ APCs

**Figure 7:**
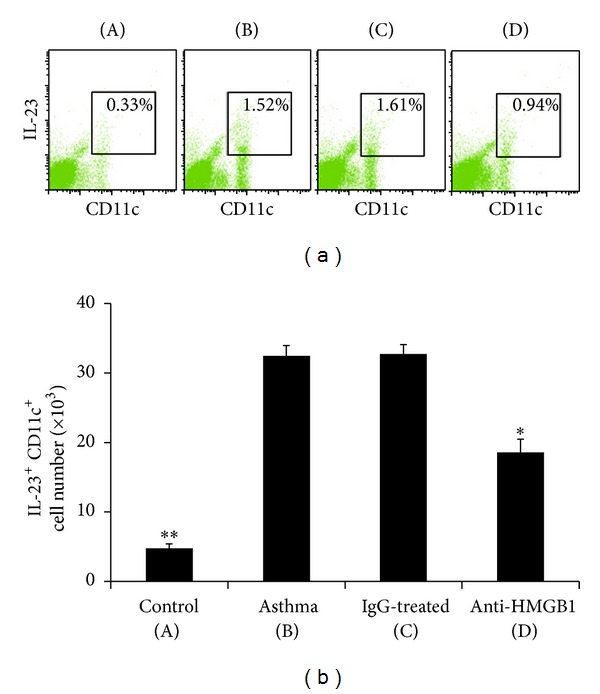
Intranasal administration of anti-HMGB1 decreased the IL-23 production by lung CD11c^+^ APCs. (a) Administration of anti-HMGB1 downregulated the percentage of IL-23^+^ CD11c^+^ APCs in low-density lung cells. Representative results of the percentage of IL-23^+^ CD11c^+^ APCs in lung from mice in the control (A), asthma (B), control IgG-treated (C), and anti-HMGB1-treated groups (D) were analyzed using FACS. (b) Histogram showing the number of IL-23^+^ CD11c^+^ APCs in mice from the different groups. The values represent the means ± standard deviations (*n* = 5). *P < 0.05 and **P < 0.01 compared with the asthma group.

To elucidate the mechanisms of the suppressive Th17 response, we examined the effect of anti-HMGB1 IgG on lung APC functions. Consistent with the marked reduced level of IL-23 in the BALF, the administration of anti-HMGB1 IgG also significantly suppressed the production of IL-23, which is an important cytokine that is used to differentiate and promote the Th17 response [24], by lung CD11c^+^ APCs (*P* < 0.05 versus Asthma group or IgG group, Figure 7). These results demonstrate that anti-HMGB1 IgG could suppress the production of IL-23 by lung CD11c^+^ APCs, thus inhibiting the development of the Th17-mediated immune response in the lung.

### 3.7. Th17 Priming Induced by i.t. Injection of HMGB1-Stimulated BMDCs

**Figure 8:**
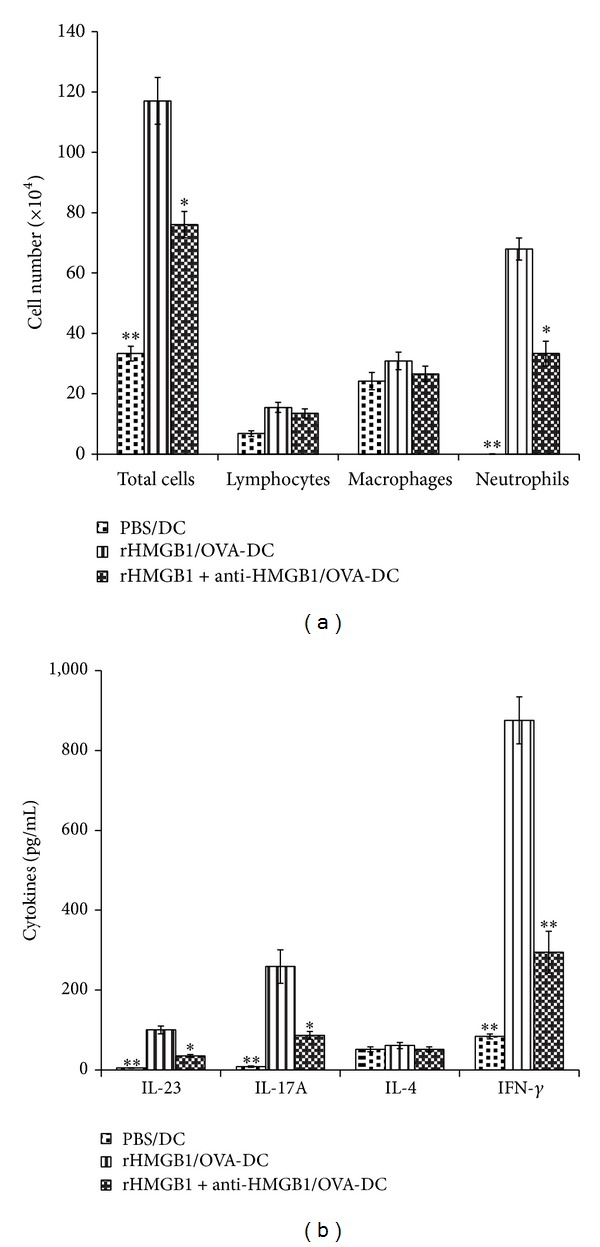
Anti-HMGB1 impaired the ability of HMGB1-stimulated BMDCs to prime Th17 responses* in vivo*. Mice received an intratracheal (i.t.) injection of 2 × 10^6^ PBS-treated, nonpulsed DCs (PBS/DCs), rHMGB1-treated, OVA-pulsed DCs (rHMGB1/OVA-DCs), or rHMGB1 plus anti-HMGB1-treated, OVA-pulsed DCs (rHMGB1 + anti-HMGB1/OVA-DCs). The mice were then exposed to OVA aerosols on days 10–12. (a) The total and differential cells counts and (b) cytokine profiles in the BALF were assessed 24 h after the final OVA exposure. The values represent the means ± standard deviations (*n* = 5). *P < 0.05 and **P < 0.01 compared with the asthma group.

To clarify the impact of HMGB1 on the potential of DCs to promote Th17 skewing* in vivo*, we adoptively transferred rHMGB1 or rHMGB1 plus anti-HMGB1 IgG-treated OVA-DCs into the airways of mice. As expected, rHMGB1/OVA-DC group mice showed a marked neutrophilic inflammation that was characterized by infiltration of neutrophils into the BALF (*P* < 0.01 versus PBS/DCs group, Figure 8(a)) and Th17/Th1 priming, as shown by the increase in IL-23, IL-17A, and IFN-*γ* levels in the BALF (*P* < 0.01 versus PBS/DCs group, Figure 8(b)). However, rHMGB1 plus anti-HMGB1 IgG pretreatment before transferring the OVA-DCs significantly decreased the neutrophils in the BALF (*P* < 0.05 versus rHMGB1/OVA-DCs group, Figure 8(a)) and the Th17 response, as shown by the decrease in the IL-23 and IL-17A levels (*P* < 0.05 versus rHMGB1/OVA-DCs group, Figure 8(b)). The above data confirmed that rHMGB1 pretreatment promoted the capability of OVA-DCs to induce neutrophilic inflammation and Th17 skewing in asthmatic mice.

## 4. Discussion

High mobility group box 1 (HMGB1) is a DNA binding, nuclear and cytosolic protein; in the extracellular environment, HMGB1 is an important mediator of inflammation that regulates tissue response to infection and injury [25]. In particular, HMGB1 controls the function of migratory cells from various origins [26, 27]. Potential sources of environmental HMGB1 are dying cells or bone marrow-derived APCs [28, 29], which express HMGB1 in the nucleus and secrete it in response to inflammatory stimuli. Manfredi reported that the receptor for advanced glycation end products (RAGE), which is a multiligand member of the Ig superfamily of cell-surface molecules that acts as a receptor for HMGB1, plays a nonredundant role in DC function [30].

Our recent studies showed that HMGB1 expression and the IL-17 level in lungs increased in a murine model of neutrophilic asthma, suggesting a possible association of the inflammatory lipid mediator HMGB1 with Th17 responses. In the present study, we provide evidence that HMGB1 could effectively modulate Th17 responses. First, we found that rHMGB1 induced increased expression of IL-23 in BMDCs, which is reported to be crucial for Th17 development. Second, we observed that TCR-activated T cells in coculture with rHMGB1-stimulated BMDCs developed a Th17 phenotype with increased expression of IL-17A. Third, our findings demonstrate that addition of anti-IL-23 into cocultures abrogated the Th17 response that was induced by rHMGB1-stimulated DCs. Taken together, these observations suggest that generation of Th17 in this BMDC-T cell coculture system is dependent upon the production of the Th17-polarizing cytokine IL-23 by BMDCs in response to rHMGB1.

In addition, we evaluated the effect of anti-HMGB1 IgG on HMGB1 expression, airway inflammation, and AHR in the murine model of neutrophilic asthma. The results showed that administration of anti-HMGB1 IgG before sensitization resulted in decreased HMGB1 expression, low levels of neutrophilic inflammation in lung tissue, little infiltration of the BALF by neutrophils, and decreased AHR. As some studies have shown that Th17 cells mediate neutrophilic airway inflammation and airway hyperresponsiveness in mice [31]; therefore, we postulated that induction of Th17 polarization by HMGB1 might represent a critical initiating event that results in neutrophilic airway inflammation and AHR. In our study, administration of anti-HMGB1 IgG resulted in decreased levels of Th17-related cytokines (IL-23 and IL-17A) in the BALF and little infiltration of the lung tissue by Th17 cells, suggesting that anti-HMGB1 treatment has an inhibitory effect on the Th17 response. To further explore the mechanism underlying the Th17 skewing effects of HMGB1, we focused on pulmonary DCs, which play a major role in the pathogenesis of asthma and preferentially express RAGE [32], which is a receptor for HMGB1. Because IL-23, which is a Th17-inducing factor, is usually produced by DCs and macrophages in lung and are sufficient to induce de novo IL-17 production from naïve CD4^+^ T cells [10, 33], we assessed the number of IL-23-producing CD11c^+^ APCs in lung cells. Consistent with the marked reduced level of IL-23 in the BALF, administration of anti-HMGB1 inhibited the number of IL-23-producing CD11c^+^ APCs in the lung, suggesting that modulation of DC function, including IL-23 production, would be a major mechanism of Th17 suppression by anti-HMGB1 IgG.

Last, our study demonstrated that HMGB1's involvement in Th17 polarization in the airways of mice is mediated, at least in part, by airway DCs. Because RAGE is expressed on DCs, monocytes, smooth muscle cells, and endothelial progenitor cells [34], HMGB1 may have direct activation effects on effector cells other than DCs during allergic inflammation. To prove that HMGB1 promotes Th17 polarization predominantly by regulating DCs, we investigated the effects of adoptive transfer of rHMGB1-treated, OVA-pulsed DCs (rHMGB1/OVA-DCs) on airway inflammation in naive mice. As expected, adoptive transfer of rHMGB1/OVA-DCs significantly induced neutrophil airway infiltration and a strong Th17 skewing response that was characterized by the release of Th17-related cytokines (IL-23 and IL-17A) in the BALF. However, the adoptive transfer of rHMGB1 plus anti-HMGB1 IgG-treated OVA-DCs (rHMGB1 + anti-HMGB1/OVA-DCs) significantly reduced neutrophil infiltration and Th17-related cytokine secretion in the BALF. These results strongly support the important role of HMGB1 signaling in DC-primed allergic disease and demonstrate that a novel role of HMGB1 is as a regulator of neutrophilic inflammation and the Th17-mediated immune response in airways.

## 5. Conclusion

In the present work, we showed that HMGB1 can contribute to the differentiation of activated T cells into Th17 cells by inducing BMDCs to produce IL-23* in vitro* and that it acts as an immune regulator of the Th17-mediated response* in vivo*. Anti-HMGB1 IgG suppressed neutrophilic inflammation, AHR, and the Th17-mediated immune response in the lung, at least partly by inhibiting the production of IL-23 by lung APCs. Therefore, antagonists of HMGB1 might be promising therapeutic molecules that could treat severe asthma characterized by dominant Th17 phenotypes.
